# 

**Published:** 1884-03

**Authors:** 


### BRISTOL MEDICO-CHIRURGICAL JOURNAL, MARCH.—1884.

### CONTENTS.

ORIGINAL PAPERS:— *Page.*

On Flat-foot and its Cure by Operation. By ALEXANDER OGSTON, C.M. ... ... 1

The Effects of certain Anatomical Relations. By JAMES CANTLIE, M.A., M.B., F.R.C.S. ... 21

On Giving Rest to the Digestive Organs by the Use of Peptonised Food. By W. H. SPENCER, M.A., M.D. ... 32

CLINICAL RECORDS:—

Two Cases of Gastric Ulcer treated by Peptonised Enemata. By Drs. SHAW and SPENCER ... 41

Case of Spontaneous Cure of Spina Bifida, followed by Hydrocephalus. By E. LONG FOX, M.D., F.R.C.P. ... 45

Case of Rupture of the Right Auricle of the Heart. By GEORGE THOMPSON, M.D. 49

Case of Successful Immediate Suturing of the Olecranon for Simple Fracture. By W. P. KEALL, M.R.C.S. ... 50

Case of Death from Fat Embolism after Bone-setting. By A. W. PRICHARD, M.R.C.S. ... 54

Case of Diphtheria treated by Tracheotomy, Peptonised Enemata and Iodoform. By R. SHINGLETON SMITH, M.D., M.R.C.P. ... 61

NOTES ON BOOKS ... 63

MEDICAL EXTRACTS ... 70

